# Increased Risk of Hospitalization for Pancreatic Cancer in the First 8 Years after a Gestational Diabetes Mellitus regardless of Subsequent Type 2 Diabetes: A Nationwide Population-Based Study

**DOI:** 10.3390/cancers13020308

**Published:** 2021-01-15

**Authors:** Julien Simon, Karine Goueslard, Patrick Arveux, Sonia Bechraoui-Quantin, Jean-Michel Petit, Catherine Quantin

**Affiliations:** 1 High-Dimensional Biostatistics for Drug Safety and Genomics, Inserm U1018 Center of Research in Epidemiology and Population Health (CESP), Université Paris-Saclay, 94800 Villejuif, France; simonjulien@free.fr; 2 Biostatistics and Bioinformatics (DIM), University Hospital, 21079 Dijon, France; karine.goueslard@chu-dijon.fr (K.G.); s.bechraouiquantin@student.helmo.be (S.B.-Q.); 3 Center for Primary Care and Public Health, Unisanté, University of Lausanne, 1003 Lausanne, Switzerland; Patrick.Arveux@unisante.ch; 4 Department of Endocrinology, Diabetes and Metabolic Disorders, Dijon University Hospital, 21079 Dijon, France; jean-michel.petit@chu-dijon.fr; 5 INSERM Unit, LNC-UMR 1231, University of Burgundy, 21078 Dijon, France; 6 Clinical Epidemiology/Clinical Trials Unit, Clinical Investigation Center, Inserm, CIC 1432, Dijon University Hospital, 21000 Dijon, France; 7 CHU de Dijon, Service de Biostatistique et d’Informatique Médicale, BP 77908, CEDEX 21079 Dijon, France

**Keywords:** pancreas cancer, gestational diabetes mellitus, type 2 diabetes, postpartum follow-up, medico-administrative database, national study

## Abstract

**Simple Summary:**

Pancreatic cancer is one of the cancers with the highest mortality rate which is most often attributed to late diagnosis. The identification of risk factors is therefore important. While gestational diabetes also shares mechanisms of action with diabetes mellitus, several studies have provided hypotheses that could explain the pathophysiology of the link between diabetes mellitus and risk of pancreatic cancer. Accordingly, the aim of our study was to determine the risk of developing pancreatic cancer in women with a history of gestational diabetes from a quasi-exhaustive national medico-administrative database for deliveries in France. We included 1,368,755 women in our study. We showed that gestational diabetes was significantly associated with a greater risk of pancreatic cancer, regardless of subsequent type 2 diabetes. Our results suggest a better follow-up of patients after a gestational diabetes in order to identify high-risk profiles of developing more serious conditions, such as pancreatic cancer.

**Abstract:**

The aim of this large retrospective cohort study was to use a quasi-exhaustive national medico-administrative database of deliveries in France to determine the risk of developing pancreatic cancer (PC) in women with a history of gestational diabetes mellitus (GDM). This nationwide population-based study included women aged 14–55 who gave birth between 1st January 2008 and 31 December 2009. The women were followed-up epidemiologically for eight years. Survival analyses using Cox regression models, adjusted for age, subsequent type 2 diabetes, and tobacco consumption, were performed on the time to occurrence of hospitalization for PC. The onset of GDM, tobacco consumption and subsequent type 2 diabetes were considered as time-dependent variables. Among 1,352,560 women included, 95,314 had a history of GDM (7.05%) and 126 women were hospitalized for PC (0.01%). Over the eight years of follow-up, GDM was significantly associated with a higher risk of hospitalization with PC in the first Cox regression model adjusted for age and subsequent type 2 diabetes (HR = 1.81 95% CI [1.06–3.10]). The second Cox regression model adjusted for the same covariates, plus tobacco consumption, showed that GDM was still significantly associated with a higher risk of hospitalization for PC with nearly the same estimated risk (HR = 1.77 95% CI [1.03–3.03]). Gestational diabetes was significantly associated with a greater risk of hospital admission for pancreatic cancer within eight years, regardless of subsequent type 2 diabetes.

## 1. Background

Pancreatic cancer (PC) is one of the cancers with the highest mortality rate in high-income countries, with a net survival of 6–9% at five years [1,2,3]. The high mortality rate is mainly attributed to a late diagnosis, because most patients with PC remain asymptomatic until the disease reaches an advanced stage. The identification of risk factors is therefore important for the creation of appropriate public health policies.

Many studies have reported that the risk of PC increases by approximately 80% in case of type 2 diabetes [4,5,6,7,8]. Several recent studies have provided hypotheses that could explain the pathophysiology of the link between diabetes mellitus and risk of PC [9,10,11,12]: hyperinsulinemia, hyperglycemia and low-grade inflammation. Although women with gestational diabetes mellitus (GDM) have an increased risk of diabetes mellitus over the years following delivery [13,14,15], GDM also shares mechanisms of action with type 2 diabetes, including hyperglycemia due to pancreatic β-cell dysfunction against a backdrop of chronic insulin resistance.

Although several studies have explored the association between GDM and cancer [16,17,18,19], the relationship between gestational diabetes and PC remains controversial due to limited evidence, because it has been the subject of only three studies, as follows. Two studies performed in Israel by Perrin et al. and Stella et al. (2007 and 2011) concluded that GDM increases the risk of subsequent PC, with an estimated risk around 7 [20,21]. A third study by Peng et al. based on the Taiwan National Health Insurance data (2019) showed that a history of GDM was not associated with subsequent PC [22]. A recent meta-analysis (2020), including the three previous studies, concluded that GDM was not associated with a risk of PC [23]. However, the results of the three studies should be taken with caution, because the diagnosis of GDM was not based on recent criteria in the two earlier studies and the follow-up was relatively short for some patients in the recent study. Diabetes could be considered as a symptom of pre-existing PC when its diagnosis is too close in time to the diagnosis of diabetes [24]. For this reason, we excluded PC occurring in the two years following a diagnosis of gestational diabetes. Accordingly, the aim of our study was to determine the risk of developing PC in women with a history of GDM. We used the quasi-exhaustive national medico-administrative database for deliveries in France over a 2-year inclusion period and a follow-up period of 8 years.

## 2. Methods

### 2.1. Data Sources and Study Design

We conducted a nationwide population-based retrospective cohort study of women who gave birth in France between 1st January 2008 and 31 December 2009. To this aim, we used the Système National des Données de Santé (SNDS), which is the French national health data system that contains individual, exhaustive and linkable but anonymous data on health expenditures for about 99% of the French population [25]. The SNDS pools data from the following sources: the database of the National Inter-Regime Information System on Health Insurance (Système National d’Information Inter Régimes de l’Assurance Maladie, SNIIRAM), which contains all prescriptions for drugs fully reimbursed by the national health insurance; the database of long-term diseases (LTD), which gives access to full coverage of health expenditures by the national health insurance scheme, and the statistical database on causes of death (Base de Causes Médicales de Décès, BCMD), which includes the illness or disease that caused the death, as well as other contributing factors. The SNDS also includes data from the French national hospital database (Programme de Médicalisation des Systèmes d’Informations, PMSI), which collects principal and associated diagnoses (secondary events and current comorbidities) and procedures performed during hospital stays. The diagnoses are encoded using the International Classification of Diseases, 10th revision (ICD-10) and the procedures are encoded using the French common classification system for medical procedures (Classification Commune des Actes Médicaux, CCAM). The quality of the SNDS database is evaluated through yearly monitoring and has recently undergone validation for perinatal data [26].

Women aged 14–55 who gave birth between 1st January 2008 and 31st December 2009 were identified through the ICD-10 codes Z37 (“outcome of delivery”) as associated diagnosis and/or the procedures of deliveries in discharge abstracts from CCAM. The date of inclusion into the cohort was the date of the first delivery during the two-year inclusion period.

During the selection of study population, we excluded all women with a history of pancreatic cancer (codes C25 in the principal, associated or related diagnosis in the standardized hospital discharge abstract) during pregnancy and in the year before pregnancy. We also excluded women with a history of type 1 or 2 diabetes, who were identified using: the codes E10-E14 or O24.0-O24.3 as the principal, associated or related diagnosis in the standardized discharge abstract and/or if it was notified in the LTD and/or if at least three antidiabetic drugs or insulin (class A10 of the Anatomical, Therapeutic and Chemical classification [ATC]) or two in the event of at least one large quantity were reimbursed in the year preceding pregnancy [27]. Furthermore, women who had a diagnosis of PC within two years of pregnancy were excluded from the analysis population [24]. All of the women included for analysis were followed epidemiologically for 8 years.

### 2.2. Exposure

Exposure was defined as the presence of GDM during one pregnancy within the study period (Inclusion-2017). If a woman had more than one pregnancy, the information collected in the discharge abstracts for each pregnancy was taken into account. If a gestational diabetes code (O24.4 or O24.9) was identified in at least one of these pregnancies, the woman was considered to have GDM. To take into account the fact that GDM could occur at any time during follow-up (either during the first pregnancy or subsequent pregnancies), we considered it as a time-dependent variable (i.e., the exposure was only coded as 1 from the first-time GDM was recorded). This allowed us to consider more precisely the exact time interval between the diagnosis of GDM and the onset of PC. Women classified as “without GDM” during the entire study were those for whom a GDM code was not mentioned in any of the discharge abstracts during the epidemiological follow-up. In France, national guidelines for GDM diagnosis changed during the study period. Before 2010, GDM screening was based on a two-step procedure for all women: the first test was on venous blood glucose 1 h after ingestion of 50 g of glucose and in the event of a positive result, the second screening test was performed for oral glucose tolerance. Since 2010, new national guidelines were introduced for women with some risk factors (i.e., maternal age ≥ 35 years, BMI ≥ 25, history of diabetes in the 1st degree, personal history of GDM or macrosomia). The recommended screening for these women is fasting blood glucose at the first prenatal consultation, and if it is not performed, an oral glucose tolerance test in the second trimester.

### 2.3. Outcome

The outcome of interest was hospitalization with an ICD-10 code for PC (C25) as a principal or associated diagnosis during 8-year follow-up period. In an effort to minimize the effect of possible reverse causality on our results, we excluded women who were diagnosed with PC within two years of pregnancy [24].

### 2.4. Variables

The following explanatory variables were considered: maternal age, subsequent type 2 diabetes and tobacco consumption. The maternal age at the index delivery was categorized as <26, 26–29, 30–33 and ≥34 years. Subsequent type 2 diabetes during follow-up was defined by: the presence of one of the ICD-10 codes E10 to E14 as principal, associated or related diagnoses and/or if subsequent diabetes was notified in LTD and/or if there was a reimbursement of at least three antidiabetic drugs or insulin (class A10 of the Anatomical, Therapeutic and Chemical classification (ATC) with the exception of benfluorex) or two reimbursements if there was at least one large packaging [27]. Tobacco consumption in the year before the pregnancy, at inclusion or during follow-up was identified using the ICD-10 codes F17, T65.2, Z58.7, Z72.0, or Z71.6 in hospitalization records and/or at least one reimbursement of varenicline (Identification Codes of pharmaceutical speciality).

The time duration (days) from the delivery (with or without GDM) to the onset of the PC was determined.

Tobacco consumption was determined in two ways, as a time-dependent variable (i.e., onset) and a fixed variable (presence). The onset of tobacco consumption took into account the time interval from the moment smoking was detected (during hospitalization or following smoking cessation) in our database. The presence of tobacco consumption was determined as any smoking from the time of inclusion, regardless of when smoking was recorded in the year before the pregnancy and during the follow-up period.

### 2.5. Statistical Analyses

The characteristics of women that are categorical variables were described by the GDM groups (with GDM history and without GDM history) using frequency distributions. The two groups were compared using χ2 tests. Continuous variables, notably time durations, were described using mean (with standard deviation). The risk of hospitalization with PC was investigated using Cox proportional hazards regression models. The onset of GDM, tobacco consumption and hospitalization with subsequent type 2 diabetes were considered as time-dependent variables. We developed a first model including age, GDM, and subsequent type 2 diabetes, and a second model that adjusted for the same covariates, plus onset of tobacco consumption as a time-dependent variable. This approach may be considered questionable, as a patient may have started smoking before being identified as a smoker (by being mentioned during hospitalization or by being given a smoking cessation medication). Furthermore, a sensitivity analysis was performed on the second model, where tobacco consumption onset was replaced with the presence of tobacco consumption as a fixed variable. Hazard ratios (HR) and 95% confidence intervals (CI) were calculated in each model. We also explored the interactions between age or tobacco consumption and GDM. Finally, we ran an analysis assessing the E-value [28], which is a measure of an association’s robustness to potential uncontrolled confounders (e.g., obesity). Computer software SAS 9.4 was used for the descriptive and multivariate analyses.

This study was approved by the French national committee for data protection (registration number 12/11/2019-DR-2019-350). Individual written consent was not needed for this study. Data from the SNDS database was provided by the French National Health Insurance Fund (21/02/2019 nº 226847).

## 3. Results

**Figure 1:**
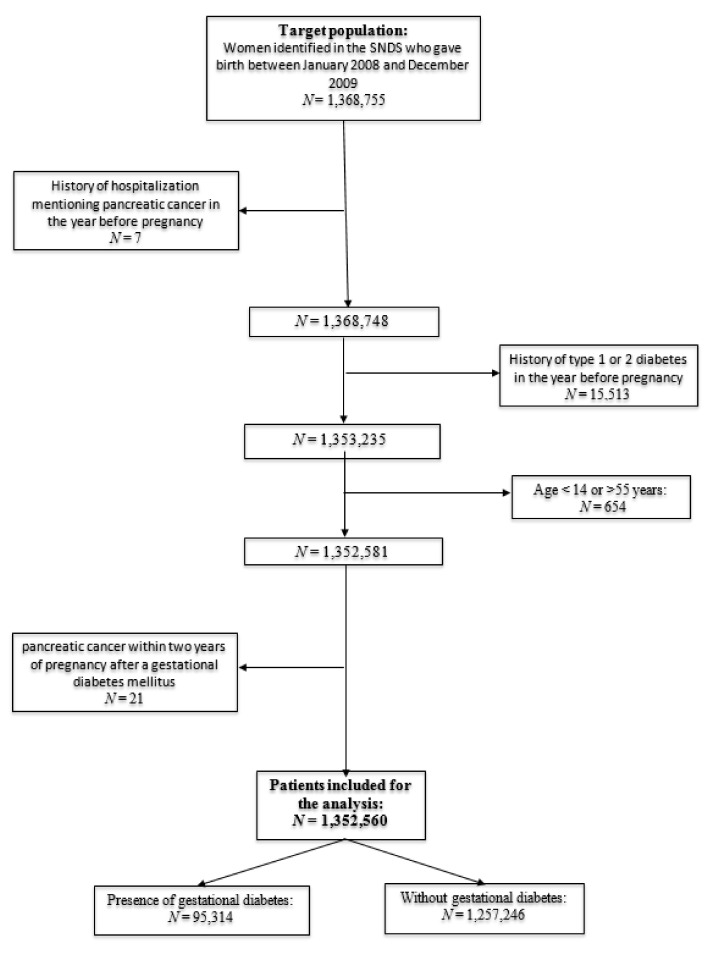
Flowchart of the study population.

From January 2008 to December 2009, 1,368,755 women hospitalized for delivery were identified in the SNDS database. Seven women who were hospitalized for PC before pregnancy and 21 women who were hospitalized for PC within 2 years after pregnancy were excluded (4 with gestational diabetes and 17 without) (Figure 1).

Similarly, 15,531 women with type 2 diabetes and 654 women who did not meet the age criteria were also excluded, leading to 1,352,560 women who were included in the analysis population. The mean duration time from the delivery to the onset of PC over all included patients was 1910 days (sd = 604). We also found that the mean duration time from the delivery of the 1st pregnancy with GDM (to compute this mean duration from the exposure with GDM) to the onset of PC was 1616 days (sd = 715) in the group with GDM and 1923 days (sd = 614) in the group without GDM.

Among the 1,352,560 women included in our study, 95,314 had GDM (7.05%) during follow-up and 126 women were hospitalized for PC (0.01%). A comparison of the characteristics of women with and without GDM is presented in Table 1.

The results of the first Cox model, which is adjusted for age and subsequent type 2 diabetes (as a time-dependent covariate), showed that GDM (as a time-dependent variable) was significantly associated with a higher risk of hospitalization with PC (HR = 1.81, 95% CI [1.06–3.10]). The results of the second Cox model adjusted for the same covariates, plus tobacco consumption (as a time-dependent covariate), showed that GDM was still significantly associated with a higher risk of hospitalization with PC with nearly the same estimated relative risk (HR = 1.77, 95% CI [1.03–3.03]). We found that hospitalization with subsequent type 2 diabetes or tobacco consumption (considered as a time-dependent variable or as a fixed variable in the sensitivity analysis) did not alter the relationship between GDM and hospitalization with PC. The interaction between tobacco consumption and GDM was non-significant (*p* = 0.60) but the interaction between age and GDM was marginally significant (*p* = 0.05). After including this interaction in Model 1, the effect of GDM was even higher. In the analysis assessing the E-value, we found that the observed HR= 1.77 could be explained away by a minimum strength of association that an unmeasured confounder must have with gestational diabetes exposure and pancreatic cancer, as low as 2.93 (Table 2).

## 4. Discussion

To our knowledge, this is the first study that specifically explores the relationship between GDM and PC, after adjustment for subsequent diabetes, which is the main potential confounding factor. In our population-based study, using a nationwide well documented retrospective cohort, we found that GDM was significantly associated with a greater risk of being hospitalized for PC within the first eight years, regardless of whether diabetes mellitus developed subsequently.

In the literature, the three studies on this subject report variable methodologies and divergent results. The two Israeli studies show similar significant relative risks, around 7, but with wide confidence intervals [20,21]. In addition, the Israeli studies were hospital-based, including patients from three hospitals in Perrin et al. and only one in Sella et al. They also found a limited number of cases: fifty-four in Perrin et al. and nine in Sella et al., compared to our study which identified 126 cases among the 1,352,560 included women. The larger population in our study explains the narrower confidence interval. In addition, our smaller relative risk may be explained by an adjustment for subsequent diabetes. Our inclusion period (2008–2009) is much more recent than in Perrin et al. (1964–1975), which was not based on recent diagnostic criteria for GDM. This may have impacted their results by creating a classification bias when selecting only the most severely exposed women. On the other hand, the Taiwanese study of Peng et al. is more comparable to our study, since it is also a population-based study using a national medico-administrative data [22]. They included nearly the same number of women (1,466,596 women) but observed a larger number of pancreatic cancer cases (331 cases). Though they found twice as many cases, they were not able to show a significant increased risk of PC in women with a history of GDM (HR = 1.07, 95% CI 0.65–1.75). However, they took into account all PCs occurring after pregnancy while we waited until two years after the occurrence of GDM to record a diagnosis of PC. In addition, it has been shown that Asian populations develop diabetes at a younger age, require insulin treatment more often, and see an increased risk of cancer compared to Western populations [29,30], which may explain the higher number of PC cases. Moreover, unlike our study, the follow-up in the Taiwanese study varied between women, some of whom were followed for more than twelve years while others were followed for only one year. Finally, we included GDM as a time-dependent variable, which might explain why we found a statistically significant result despite having fewer cases.

The first strength of our study is our adjustment for type 2 diabetes. To our knowledge, our study is unique in that it adjusts for this major potential confounding factor. Several studies have shown that there is a relationship between GDM and diabetes mellitus, and type 2 diabetes is recognized as directly or indirectly increasing the risk of PC through the degeneration of intraductal papillary mucinous neoplasms [31]. Therefore, it seemed important to consider from various aspects this confounding factor in the methodology of our study, as follows. Firstly, regarding our database, a validation study in a French cohort by Fuentes et al. showed that type 2 diabetes was identified in the national database (SNDS) with a positive predictive value of 97.2%, a sensitivity of 93.8%, and a specificity of 99.9% [32]. Secondly, we excluded women with a history of type 2 diabetes in order to refine the definition of GDM and remove the impact of the history of diabetes mellitus on the analysis. Finally, diabetes mellitus occurring during follow-up was included in our models as a time-dependent variable. By using Cox regression models, it was possible to introduce explanatory variables known at the time of inclusion but also information collected during the follow-up. This allowed us to take into account a change in status, for example, from non-diabetic to diabetic, but also the time of exposure to diabetes before hospitalization for PC.

Time is also an important consideration for the study of PC. Firstly, regarding the chronology of the disease itself, some studies show that hospitalization is a good marker for new cases for some cancers that are aggressive and/or requiring hospitalization [33,34]. Knowing that we took into account all types of hospitalizations (including diagnosis, radiotherapy, chemotherapy, surgery and follow-up), we were able to exclude subjects with a history of hospitalization for PC one year before-, during- and two years after pregnancy (i.e., almost four years). It is known that PC has a net five-year survival rate of 6–9% [1,2,3]. By eliminating women with signs of PC in the first four years, we ensured in this study that there was a very strong probability of including only incident cases of PC. Secondly, to our knowledge, ours is the only study that has established a two-year lapse of time between pregnancy (with or without GDM) and the diagnosis of PC. It has been shown that case selection should not include newly diagnosed diabetes, because it is known that a diagnosis of diabetes can be a symptom of PC. Indeed, new-onset diabetes caused by PC may be associated with pro-inflammatory alterations, insulin resistance, and perturbations in β-cell functions that lead to loss of glucose homeostasis. Adipocytes and resident macrophages release many deleterious pro-inflammatory factors, and the increase in lipolysis could enhance hepatic production of acetyl coenzyme-A, causing hepatic insulin resistance and excessive glucose release by the liver [24].

Our study has other strengths. First of all, our population-based study comprised 10 years of data, allowing us to have eight years of follow-up for all included women. It relied on a quasi-exhaustive national database of deliveries (99.6%), because almost all childbirths in France take place in hospitals and all hospitals stays are recorded in this database. In addition, in a national validation study [35], our team showed that GDM was identified in this database with a positive predictive value of 88.9%.

We also recognize that our study has some limitations. First, our recent validation study showed that sensitivity for GDM was only of 79%, which means that 21% of women with GDM were not identified as such in our database [26]. As a consequence, this classification bias may have led to an underestimation of the association. In addition, national guidelines for the diagnosis of GDM have changed from a two-step procedure in all patients before 2010, to a different (only one-step) procedure in patients with risk factors since 2010. Second, although we have a longer and more homogenous follow-up time compared to the three previous studies, eight years remains relatively short for the study of the onset of PC, most of which are diagnosed after 65 years, and we may thus have underestimated the full impact of GDM. Third, we did not have completed data on gestation and parity in our national database for the years under review. Fourth, although we included “tobacco consumption” as an adjustment variable, this information is incomplete in our administrative database. This is why we carried out two multivariate models, one with this variable and the other without; both of them provided similar results. Fifth, some other known risk factors could not be included in our study. Although we wanted to add “alcohol consumption” to our adjustment variables, since it is another well-known risk factor for pancreatic cancer, it was not feasible to do so because this variable is too poorly recorded in our database [36]. Moreover, the “obesity” variable, which is known to be associated with gestational diabetes and pancreatic cancer, could not be included in our model. Unfortunately, BMI before pregnancy is poorly documented in our hospital database and BMI during pregnancy may not be considered as reliable. This is why we conducted an analysis of the E-value, which showed that the observed HR (1.77) could be explained away by a minimum strength of association that an unmeasured confounder must have with gestational diabetes exposure and pancreatic cancer, as low as 2.93. The E-value is thus large and substantial unmeasured confounding would be necessary to cancel the observed association. In the literature, we found that obese women are 3.36 times more likely than non-obese women to have gestational diabetes [37] and 1.54 times more likely to have pancreatic cancer [38]. As both association levels are very different, we further calculated the bias B, or the largest factor by which the observed association could be reduced by the unmeasured confounder of these particular strengths. The B value calculated from these values should be higher than our HR of 1.77 to be explained by the unmeasured “obesity” variable, but it is equal to 1.33 and therefore lower than 1.77 [28]. This strengthens the conclusion derived from the E-value analysis. Sixth, additional studies with longer follow-up or including more confounding factors are needed to support these findings. In the same way, further studies are needed to investigate the relationship between GDM and survival in PC patients.

Gestational diabetes is still considered by some professionals to be a disease that has little impact once pregnancy is over, resulting in poor quality short- and long-term postpartum follow-up [39,40,41,42]. However, pregnancy is a kind of “stress-test” for the body of women, which reveals some patients’ vulnerabilities. For instance, GDM seems to be an important marker for diseases that may occur later in life. Indeed, we found in a previous study that gestational diabetes exposes women to a subsequent increased risk of type 2 diabetes mellitus, metabolic syndrome and cardiovascular conditions [43]. The results of this study suggest that an increased risk of PC should be added to this list. All these results are in favor of a better adherence to the recommendations [44,45] for following-up patients with GDM in the postpartum period. In the US, like in France, screening for type 2 diabetes is recommended during the postnatal period, and then every 1–3 years, depending on the risk factors, for at least 25 years. After GDM, health professionals should encourage lifestyle changes (30 to 60 min of daily physical activity at least five days per week, balanced diet, smoking cessation…) which are also useful for the prevention of PC [46,47]. Monitoring should also include regular screening and treatment of any other associated cardiovascular risk factors (arterial hypertension, dyslipidemia).

## 5. Conclusions

In conclusion, our study shows that gestational diabetes mellitus is a factor associated with the development of pancreatic cancer within eight years after delivery. However, the impact of gestational diabetes is overlooked by some professionals even if it exposes women to an increased risk of type 2 diabetes mellitus, metabolic syndrome and cardiovascular diseases. Our results suggest the importance of better adherence to the recommendations concerning the follow-up of patients with gestational diabetes in the postpartum period. To be effective, this follow-up would require the participation of all health professionals, including general practitioners, gynecologists, obstetricians, midwives, and endocrinologists. To render this follow-up feasible, gestational diabetes could be included among other risk factors (e.g., diabetes mellitus, obesity, metabolic syndrome, smoking or alcohol consumption) in order to identify individuals with a high risk of developing more serious conditions such as pancreatic cancer. These individuals can thus be encouraged to attend regular screening and advised about appropriate lifestyle and dietary measures.

## Figures and Tables

**Table 1:** Comparison of characteristics of women with history of GDM or without history of GDM.

Variables	GDM+ *N* = 95,314 (7.05%)		GDM− *N* = 1,257,246 (92.95%)		*p*
Age * (years)					<0.0001
<26	18,095	19.0%	299,873	23.9%	
26–29	26,013	27.3%	352,910	28.1%	
30–33	24,279	25.5%	313,954	25.0%	
≥34	26,927	28.2%	290,509	23.1%	
Hospitalization with Pancreatic Cancer	18	0.02%	108	0.01%	0.004
Subsequent type 2 diabetes	8184	8.6%	6840	0.5%	<0.0001
Tobacco consumption **	7818	8.2%	86,403	6.9%	<0.0001

*N*, number; GDM, Gestational Diabetes Mellitus; * Age between 14 and 55 years. ** Presence of tobacco consumption at any time, as used in the sensitivity analysis.

**Table 2:** Cox models of the relation between GDM and hospitalization with pancreatic cancer (n = 126). GDM, subsequent type 2 diabetes and tobacco consumption are time-dependent covariates.

Variables	Non-Adjusted		Cox Model 1 *		Cox Model 2 **	
	HR	95% CI	HR	95% CI	HR	95% CI
GDM	2.59	1.57–4.26	1.81	1.06–3.10	1.77	1.03–3.03
Age ***						
<26	0.60	0.28–1.27	0.61	0.28–1.30	0.56	0.26–1.21
26–29	1		1		1	
30–33	1.85	1.06–3.22	1.83	1.05–3.19	1.88	1.08–3.27
>34	3.77	2.28–6.23	3.60	2.18–5.96	3.67	2.22–6.08
Type 2 diabetes	8.06	3.76–17.32	4.85	2.13–11.06	4.74	2.08–10.79
Tobacco consumption	2.8	1.72–4.56	-	-	3.22	1.97–5.26

GDM: Gestational Diabetes Mellitus; HR: Hazard Ratio. * Model 1 includes GDM, age and type 2 diabetes; ** Model 2 includes GDM, age, type 2 diabetes and tobacco consumption; *** Age between 14 and 55 years.

## Data Availability

Data used in this study are available for researchers who meet the criteria for access to these French data from the National Health Insurance Fund (CNAM—Caisse nationale de l’assurance maladie).

## Abbreviations

gestational diabetes mellitus (GDM); Pancreatic cancer (PC); Système National des Données de Santé (SNDS); Système National d’Information Inter Régimes de l’Assurance Maladie (SNIIRAM); long-term diseases (LTD); Base de Causes Médicales de Décès (BCMD); Programme de Médicalisation des Systèmes d’Informations (PMSI); International Classification of Diseases, 10th revision (ICD-10); Anatomical Therapeutic and Chemical classification (ATC); Hazard Ratios (HR); Confidence Intervals (CI).

## References

[1] Drouillard A., Manfredi S., Lepage C., Bouvier A.-M. (2018). Epidemiology of pancreatic cancer. Bull. Cancer JANV.

[2] https://seer.cancer.gov/statfacts/html/pancreas.html.

[3] Rawla P., Sunkara T., Gaduputi V. (2019). Epidemiology of Pancreatic Cancer: Global Trends, Etiology and Risk Factors. World J. Oncol..

[4] Huxley R., Ansarymoghaddam A., De González A.B., Barzi F., Woodward M.J. (2005). Type-II diabetes and pancreatic cancer: A meta-analysis of 36 studies. Br. J. Cancer.

[5] Elena J.W., Steplowski E., Yu K., Hartge P., Tobias G.S., Brotzman M.J., Chanock S.J., Stolzenberg-Solomon R.Z., Arslan A.A., Bueno-De-Mesquita H.B. (2013). Diabetes and risk of pancreatic cancer: A pooled analysis from the pancreatic cancer cohort consortium. Cancer Causes Control.

[6] Chodick G., Heymann A.D., Rosenmann L., Green M.S., Flash S., Porath A., Kokia E., Shalev V. (2010). Diabetes and risk of incident cancer: A large population-based cohort study in Israel. Cancer Causes Control.

[7] Everhart J., Wright D. (1995). Diabetes mellitus as a risk factor for pancreatic cancer. A meta-analysis. JAMA.

[8] Zhou X.H., Qiao Q., Zethelius B., Pyörälä K., Söderberg S., Pajak A., Stehouwer C.D.A., Heine R.J., Jousilahti P., for the DECODE Study Group (2010). Diabetes, prediabetes and cancer mortality. Diabetologia.

[9] Biadgo B., Abebe M. (2016). Type 2 Diabetes Mellitus and Its Association with the Risk of Pancreatic Carcinogenesis: A Review. Korean J. Gastroenterol..

[10] Rahn S., Zimmermann V., Viol F., Knaack H., Stemmer K., Peters L., Lenk L., Ungefroren H., Saur D., Schäfer H. (2018). Diabetes as risk factor for pancreatic cancer: Hyperglycemia promotes epithelial-mesenchymal-transition and stem cell properties in pancreatic ductal epithelial cells. Cancer Lett..

[11] Eibl G., Cruz-Monserrate Z., Korc M., Petrov M.S., Goodarzi M.O., Fisher W.E., Habtezion A., Lugea A.P., Pandol S.J., Hart P.A. (2018). Diabetes Mellitus and Obesity as Risk Factors for Pancreatic Cancer. J. Acad. Nutr. Diet..

[12] Paternoster S., Falasca M. (2020). The intricate relationship between diabetes, obesity and pancreatic cancer. Biochim. Biophys. Acta (BBA) Bioenerg..

[13] Bellamy L., Casas J.-P., Hingorani A.D., Williams D. (2009). Type 2 diabetes mellitus after gestational diabetes: A systematic review and meta-analysis. Lancet.

[14] Kaul P., Savu A., Nerenberg K.A., Donovan L.E., Chik C.L., Ryan E.A., Johnson J.A. (2015). Impact of gestational diabetes mellitus and high maternal weight on the development of diabetes, hypertension and cardiovascular disease: A population-level analysis. Diabet. Med..

[15] Retnakaran R., Shah B.R. (2016). Role of Type 2 Diabetes in Determining Retinal, Renal, and Cardiovascular Outcomes in Women With Previous Gestational Diabetes Mellitus. Diabetes Care.

[16] Dawson S.I. (2003). Long-term risk of malignant neoplasm associated with gestational glucose intolerance. Cancer.

[17] Fuchs O., Sheiner E., Meirovitz M., Davidson E., Sergienko R., Kessous R. (2017). The association between a history of gestational diabetes mellitus and future risk for female malignancies. Arch. Gynecol. Obstet..

[18] Trabert B., Troisi R., Grotmol T., Ekbom A., Engeland A., Gissler M., Glimelius I., Madanat-Harjuoja L., Sørensen H.T., Tretli S. (2020). Associations of pregnancy-related factors and birth characteristics with risk of endometrial cancer: A Nordic population-based case–control study. Int. J. Cancer.

[19] Tong G.-X., Cheng J., Chai J., Geng Q.-Q., Chen P.-L., Shen X.-R., Liang H., Wang D. (2014). Association Between Gestational Diabetes Mellitus and Subsequent Risk of Cancer: A Systematic Review of Epidemiological Studies. Asian Pac. J. Cancer Prev..

[20] Perrin M.C., Terry M.B., Kleinhaus K.R., Deutsch L., Yanetz R., Tiram E., Calderon-Margalit R., Friedlander Y., Paltiel O., Harlap S. (2007). Gestational diabetes as a risk factor for pancreatic cancer: A prospective cohort study. BMC Med..

[21] Sella T., Chodick G., Barchana M., Heymann A.D., Porath A., Kokia E., Shalev V. (2011). Gestational diabetes and risk of incident primary cancer: A large historical cohort study in Israel. Cancer Causes Control.

[22] Peng Y.-S., Lin J.-R., Cheng B.-H., Ho C., Lin Y.-H., Shen C.-H., Tsai M.-H. (2019). Incidence and relative risk for developing cancers in women with gestational diabetes mellitus: A nationwide cohort study in Taiwan. BMJ Open.

[23] Wang Y., Yan P., Fu T., Yuan J., Yang G., Liu Y., Zhang Z.-J. (2020). The association between gestational diabetes mellitus and cancer in women: A systematic review and meta-analysis of observational studies. Diabetes Metab..

[24] Pannala R., Leirness J.B., Bamlet W.R., Basu A., Petersen G.M., Chari S.T. (2008). Prevalence and Clinical Profile of Pancreatic Cancer–Associated Diabetes Mellitus. Gastroenterology.

[25] Tuppin P., Rudant J., Constantinou P., Gastaldi-Ménager C., Rachas A., de Roquefeuil L., Maura G., Caillol H., Tajahmady A., Coste J. (2017). Value of a national administrative database to guide public decisions: From the système national d’information interrégimes de l’Assurance Maladie (SNIIRAM) to the système national des données de santé (SNDS) in France. Rev. Epidemiol. Sante Publique.

[26] Goueslard K., Cottenet J., Benzenine E., Tubert-Bitter P., Quantin C. (2020). Validation study: Evaluation of the metrological quality of French hospital data for perinatal algorithms. BMJ Open.

[27] Fosse-Edorh S., Rigou A., Morin S., Fezeu L., Mandereau-Bruno L., Fagot-Campagna A. (2017). Algorithms based on medico-administrative data in the field of endocrine, nutritional and metabolic diseases, especially diabetes. Rev. Epidemiol. Sante Publique.

[28] VanderWeele T.J., Ding P. (2017). Sensitivity Analysis in Observational Research: Introducing the E-Value. Ann. Intern. Med..

[29] Ma R.C., Chan J.C. (2013). Type 2 diabetes in East Asians: Similarities and differences with populations in Europe and the United States. Ann. N. Y. Acad. Sci..

[30] Noto H., Tsujimoto T., Noda M. (2011). Significantly increased risk of cancer in diabetes mellitus patients: A meta-analysis of epidemiological evidence in Asians and non-Asians. J. Diabetes Investig..

[31] Pergolini I., Schorn S., Jäger C., Goess R., Novotny A., Friess H., Ceyhan G.O., Demir I.E. (2020). Diabetes mellitus in intraductal papillary mucinous neoplasms: A systematic review and meta-analysis. Surgery.

[32] Fuentes S., Cosson E., Mandereau-Bruno L., Fagot-Campagna A., Bernillon P., Goldberg M., Fosse-Edorh S., CONSTANCES-Diab Group (2018). Identifying diabetes cases in health administrative databases: A validation study based on a large French cohort. Int. J. Public Health.

[33] Bousquet P.-J., Caillet P., Coeuret-Pellicer M., Goulard H., Kudjawu Y.C., Le Bihan C., Lecuyer A., Séguret F. (2017). Using cancer case identification algorithms in medico-administrative databases: Literature review and first results from the REDSIAM Tumors group based on breast, colon, and lung cancer. Rev. Epidemiol. Sante Publique.

[34] Wu J.W., Azoulay L., Huang A., Paterson M., Wu F., Secrest M.H., Filion K.B. (2020). Identification of incident pancreatic cancer in Ontario administrative health data: A validation study. Pharmacoepidemiol. Drug Saf..

[35] Pierron A., Revert M., Goueslard K., Vuagnat A., Cottenet J., Benzenine E., Fresson J., Quantin C. (2015). Evaluation of the metrological quality of the medico-administrative data for perinatal indicators: A pilot study in 3 university hospitals]. Rev. Epidemiol. Sante Publique.

[36] Yadav D., Lowenfels A.B. (2013). The Epidemiology of Pancreatitis and Pancreatic Cancer. Gastroenterology.

[37] Torloni M.R., Betrán A.P., Horta B.L., Nakamura M.U., Atallah A.N., Moron A.F., Valente O. (2009). Prepregnancy BMI and the risk of gestational diabetes: A systematic review of the literature with meta-analysis. Obes. Rev..

[38] Johansen D., Stocks T., Jonsson H., Lindkvist B., Björge T., Concin H., Almquist M., Häggström C., Engeland A., Ulmer H. (2010). Metabolic Factors and the Risk of Pancreatic Cancer: A Prospective Analysis of almost 580,000 Men and Women in the Metabolic Syndrome and Cancer Project. Cancer Epidemiol. Biomark. Prev..

[39] Goueslard K., Cottenet J., Mariet A.-S., Sagot P., Petit J.-M., Quantin C. (2017). Early screening for type 2 diabetes following gestational diabetes mellitus in France: Hardly any impact of the 2010 guidelines. Acta Diabetol..

[40] Quaresima P., Visconti F., Chiefari E., Puccio L., Foti D., Venturella R., Vero R., Brunetti A., Di Carlo C. (2018). Barriers to Postpartum Glucose Intolerance Screening in an Italian Population. Int. J. Environ. Res. Public Health.

[41] Boyle D.I., Versace V.L., Dunbar J., Scheil W., Janus E., Oats J.J.N., Skinner T.C., Shih S., O’Reilly S., Sikaris K. (2018). Results of the first recorded evaluation of a national gestational diabetes mellitus register: Challenges in screening, registration, and follow-up for diabetes risk. PLoS ONE.

[42] Bernstein J.A., Quinn E., Ameli O., Craig M., Heeren T., Iverson R., Jack B., Lee-Parritz A., McCloskey L. (2018). Onset of T2DM after gestational diabetes: What the prevention paradox tells us about risk. Prev. Med..

[43] Goueslard K., Cottenet J., Mariet A.-S., Giroud M., Cottin Y., Petit J.-M., Quantin C. (2016). Early cardiovascular events in women with a history of gestational diabetes mellitus. Cardiovasc. Diabetol..

[44] Collège National des Gynécologues et Obstétriciens Français, Société Francophone du Diabète (2010). Gestational diabetes. J. Gynecol. Obstet. Biol. Reprod.

[45] American Diabetes Association (2020). 14.Management of Diabetes in Pregnancy: Standards of Medical Care in Diabetes—2020. Diabetes Care.

[46] Capasso M., Franceschi M., Rodriguez-Castro K.I., Crafa P., Cambiè G., Miraglia C., Barchi A., Nouvenne A., Leandro G., Meschi T. (2018). Epidemiology and risk factors of pancreatic cancer. Acta Biomed..

[47] Midha S., Chawla S., Garg P.K. (2016). Modifiable and non-modifiable risk factors for pancreatic cancer: A review. Cancer Lett..

